# Intratumoral and Peritumoral Radiomics based on Contrast-enhanced CT Images: Biomarkers to Predict the Risk-Grade of Gastrointestinal Stromal Tumors

**DOI:** 10.2174/0115734056394776251126063546

**Published:** 2026-02-02

**Authors:** Yiming Liu, Ying Wu, Xinyue Cao, Guojin Zhang, Longlin Yin

**Affiliations:** 1 Department of Radiology, Sichuan Provincial People's Hospital, Wenjiang Hospital, Chengdu, China; 2 Department of Radiology, Sichuan Provincial People's Hospital, University of Electronic Science and Technology of China, Chengdu, China; 3 Institute of Radiation Medicine, Sichuan Provincial People's Hospital, University of Electronic Science and Technology of China, Chengdu, China

**Keywords:** Gastrointestinal stromal tumor, Radiomics, Computed tomography, Rad-score model, SHAP, Low-risk and high-risk groups, Concurrent gastrointestinal tract tumors

## Abstract

**Introduction:**

This study aimed to investigate the value of intratumoral and peritumoral radiomics in predicting the risk grade of gastrointestinal stromal tumors (GISTs) using contrast-enhanced computed tomography (CT) images.

**Methods:**

A total of 217 pathology-confirmed GISTs were retrospectively enrolled and divided into low-risk and high-risk groups. Significant predictors were selected from clinical and radiological characteristics to build a prediction model. Radiomics features were extracted from the intratumoral region, the 3-mm peritumoral region, and the 5-mm peritumoral region. After ANOVA and LASSO feature screening, logistic regression was applied to construct the radiomics model. The Rad-score of the optimal radiomics model was calculated and combined with the selected radiological characteristics to develop a combined model and a nomogram. ROC curves were used to assess the predictive performance of each model, while calibration curves and decision curve analysis were used to evaluate their clinical utility. The SHapley Additive Explanations (SHAP) method was applied to perform interpretability analysis of the optimal model.

**Results:**

A radiological model (RM), five radiomics models, and a combined radiological characteristics plus Rad-score model (CRM) were constructed. In the validation set, the AUCs of the RM and CRM were 0.839 and 0.924, respectively. The intratumoral plus 3-mm peritumoral radiomics model (ITV+PTV3) achieved the best performance in the validation set, with an AUC of 0.934.

**Discussion:**

The ITV+PTV3 model shows strong potential for objective GIST risk stratification but requires multi-center prospective validation to ensure generalizability beyond the limitations of this retrospective dataset.

**Conclusion:**

Radiomics models based on intratumoral and peritumoral regions perform well in predicting the risk grade of GISTs and may effectively guide accurate preoperative diagnosis and treatment planning.

## 1 INTRODUCTION

Gastrointestinal stromal tumors (GISTs) are the most common mesenchymal tumors of the digestive system and can occur anywhere within it [1]. Their biological behavior varies, and all GISTs possess malignant potential; therefore, they cannot be simply categorized as benign or malignant tumors [2]. The National Institutes of Health (NIH) classifies GISTs as very low-risk, low-risk, intermediate-risk, and high-risk based on histopathological examination, according to maximum tumor diameter, mitotic count, tumor site, and presence of tumor rupture [3].

Treatment options differ according to risk grade. Currently, surgical resection is the main treatment strategy for very low-risk and low-risk patients, while intermediate-risk and high-risk patients typically require surgical resection combined with adjuvant targeted therapy, such as imatinib [4]. Therefore, a reliable risk-classification system is essential for assessing the biological behavior and prognosis of GISTs and for guiding treatment decisions.

Despite their widespread use, current risk assessment methods for GISTs have several limitations. First, histopathological examination, the current gold standard, may lead to tumor rupture, bleeding, or even metastasis due to the fragile nature of GISTs [5, 6]. Additionally, tumor heterogeneity can result in an inaccurate representation of malignancy when only a small tissue sample is analyzed. Moreover, computed tomography (CT), the standard non-invasive preoperative diagnostic tool for GISTs, can reflect morphological characteristics and biological information such as tumor blood supply and may serve as a reference for risk classification; however, CT interpretation is subjective, lacks quantitative features, and shows poor reproducibility [7, 8].

Consequently, a major challenge remains: accurately and objectively evaluating the malignancy of GISTs preoperatively using a non-invasive method.

Radiomics can extract deep imaging information and uncover a large amount of micro-level biological data that is not visible to the naked eye, offering the advantages of being non-invasive, quantitative, and reproducible [9, 10]. It has been widely applied in tumor diagnosis, treatment planning, and prognosis evaluation [11-13]. Several studies have shown that combining radiomics with clinical and radiological features can further enhance model performance [14]. Recent research has demonstrated that radiomics based on CT images can capture information often overlooked in traditional CT interpretation, thereby improving the accuracy and objectivity of GIST risk assessment with relatively high efficacy [15, 16].

However, these studies have primarily focused on the intratumoral region, while the value of the peritumoral region in GISTs has been largely overlooked. Higher-risk GISTs may invade adjacent structures or even rupture [17], yet subtle changes in the peritumoral region on CT images may be difficult to detect visually. Previous research has confirmed that radiomics models incorporating peritumoral regions can better reflect tumor biological behavior [18, 19]. Moreover, studies have shown that GISTs typically exhibit progressive enhancement on contrast-enhanced CT, and lesions are most clearly visualized during the venous phase when CT values approach their peak, which facilitates accurate segmentation [20].

Because radiomics methods can be complex and abstract, the Shapley Additive Explanations (SHAP) technique has been introduced to help avoid “black box” models. SHAP quantifies the contribution of each feature to the model’s prediction, thereby improving transparency and reliability in clinical applications [21].

In this study, we aimed to develop multiple radiomics models and a combined model to predict GIST risk classification using intra- and peritumoral regions on venous-phase CT images. We compared the diagnostic performance of different models and investigated whether the peritumoral region could more effectively predict GIST risk classification. Additionally, with the assistance of SHAP, we interpreted and visualized the models to explore a new approach for identifying GIST risk.

## 2 MATERIALS AND METHODS

### 2.1 Patients

This retrospective study was approved by the Institutional Review Board of our hospital, and the requirement for informed consent was waived. Between January 2017 and January 2022, the clinical, pathological, and CT data of patients with pathologically confirmed GIST were collected. The inclusion criteria were as follows: (1) abdominal contrast-enhanced CT performed within 30 days before surgery; (2) complete clinical, pathological, and CT data with a clearly defined risk classification; and (3) maximum tumor diameter >1 cm on CT images, allowing clear lesion delineation. The exclusion criteria were: (1) receipt of adjuvant therapy before CT examination or pathological sampling; (2) poor-quality CT images; and (3) presence of other concurrent gastrointestinal tract tumors.

A total of 217 patients (116 males and 101 females; mean age ± standard deviation, 59.4 ± 12.9 years) met the selection criteria and were randomly assigned to either the training set (152 cases) or the validation set (65 cases) in a 7:3 ratio. The detailed patient selection process is illustrated in Fig. (**1**).

Tumor risk classification was based on the NIH criteria (2008 version) [3], as shown in Table **S1**. For this study, the very low-risk and low-risk categories were combined into the low-risk group, while the intermediate-risk and high-risk categories were combined into the high-risk group.

The study followed the Sex and Gender Equity in Re search guidelines (SAGER) to ensure appropriate inclusion and reporting of sex-related data.

### 2.2 Clinical and Radiological Characteristics Analysis

The clinical characteristics included sex, age, and the presence or absence of digestive tract bleeding. Two radiologists (with 7 and 8 years of radiology experience, respectively) jointly evaluated the radiological characteristics of the tumors, including location (stomach or non-stomach), maximum diameter (measured on multiplanar reconstruction), shape (regular or irregular), boundary (clear or unclear), growth pattern (endogenous, exogenous, or mixed), and the presence of ulceration, calcification, or necrosis. Detailed definitions of these radiological characteristics are provided in the Supplementary Materials. In cases of disagreement, a senior radiologist with 28 years of experience made the final decision. All radiologists were blinded to the pathological results.

Predictors associated with GIST risk classification were initially identified through univariate analysis of the clinical and radiological characteristics. Variables showing a significant association (*p* < 0.05) in the univariate analysis were subsequently entered into a multivariable binary logistic regression model. To select robust predictors and avoid overfitting, stepwise backward variable selection was performed based on the Akaike Information Criterion (AIC). Potential multicollinearity was evaluated using variance inflation factors (VIF), with VIF values >5 indicating significant collinearity. The final model was constructed using the variables retained after this selection process.

### 2.3 CT Examination

To prepare the gastrointestinal tract, patients fasted for 6–8 hours before the CT scan. The scanning range extended from the diaphragmatic plane to the level of the umbilicus or pubic symphysis. Two 64-row spiral CT scanners (SOMATOM Definition AS+ or SOMATOM Perspective, Siemens Healthineers, Erlangen, Germany) were used, with the following parameters: tube voltage of 120 or 130 kVp; automatic tube current modulation (CARE DOSE 4D); scan layer thickness and spacing of 5 mm or 8 mm; reconstruction layer thickness of 1 mm or 1.5 mm; and reconstruction interval of 1 mm.

A non-ionic iodine contrast agent (Ultravist Iohexol 300 mg/mL; Bayer Healthcare, Leverkusen, Germany) was injected *via* the antecubital vein using a high-pressure syringe at a rate of 2.5–3.0 mL/s, with the total injection volume calculated as 1.2–1.5 mL per kilogram of body weight. Contrast-enhanced arterial and venous phase images were obtained with average delays of 25 seconds and 60 seconds after contrast administration, respectively.

### 2.4 3D Segmentation and Feature Extraction of Images

A flowchart of the radiomics analysis conducted in this study is shown in Fig. (**2**). Thin-slice venous-phase CT images of all included cases were imported into 3D Slicer software (version 4.13.0). The region of interest (ROI) covering the entire tumor was segmented slice-by-slice by a radiologist (Reader A, with 4 years of radiology experience). Intratumoral ROIs were manually delineated along the tumor boundary. Peritumoral ROIs were generated by automatically dilating the intratumoral ROIs by 3 mm and 5 mm, subtracting the intratumoral ROIs, and then manually adjusting the contours to avoid adjacent organs (such as the liver or pancreas) and blood vessels.

To assess reproducibility and stability, CT images of 20 randomly selected patients were re-segmented by another radiologist (Reader B, also with 4 years of radiology experience), and the inter-observer correlation coefficient (ICC) was calculated. A senior radiologist (with 28 years of experience) provided final confirmation whenever uncertainties arose during segmentation. Gas and gastrointestinal contents in or around the lesions were avoided during ROI delineation. The ROIs included any areas of necrosis, calcification, or ulceration within the tumor. A schematic diagram of the segmentation process is shown in Fig. (**S1A**–**F**).

Radiomics feature extraction from the intratumoral, 3-mm peritumoral, and 5-mm peritumoral regions was performed using Python (version 3.7.1) with the PyRadiomics package (pyradiomics.readthedocs.io/en/latest/index.html), following the recommendations of the International Symposium on Biomedical Imaging (ISBI) [22]. Image preprocessing included voxel resampling to 1 × 1 × 1 mm^3^ and grayscale discretization with a bin width of 25. To extract a broader set of features, the original images were further processed using the Laplacian of Gaussian (LoG) and wavelet filters. The extracted features included: (1) first-order features; (2) shape features; and (3) texture features, comprising Gray Level Dependence Matrix (GLDM), Gray Level Size Zone Matrix (GLSZM), Gray Level Run Length Matrix (GLRLM), Gray Level Co-occurrence Matrix (GLCM), and Neighborhood Gray Tone Difference Matrix (NGTDM).

### 2.5 Feature Selection and Radiomics Model Establishment

R (version 26.0) and Python (version 3.7.1) were used for radiomics feature selection and model construction. Radiomics features with ICC > 0.75 were retained, indicating satisfactory inter-observer agreement [23]. Features extracted from the 3-mm and 5-mm peritumoral regions were each combined with the intratumoral feature set, resulting in five feature matrices: intratumoral features, 3-mm peritumoral features, 5-mm peritumoral features, intratumoral + 3-mm peritumoral features, and intratumoral + 5-mm peritumoral features. All radiomics features were standardized using Z-score normalization.

Radiomics models were constructed using the training set according to the following steps: the Synthetic Minority Oversampling Technique (SMOTE) was applied to balance the proportion of low-risk and high-risk cases to approximately 1:1; ANOVA was performed to select statistically significant features; least absolute shrinkage and selection operator (LASSO) regression with 5-fold cross-validation was used to further screen features with non-zero coefficients; and logistic regression was used to establish the radiomics models. These included an intratumoral model (ITV), two peritumoral models (PTV3 and PTV5), and two combined intratumoral –peritumoral models (ITV+PTV3 and ITV+PTV5).

All models were then applied to the independent validation set to assess predictive performance, and the optimal radiomics model was selected for Rad-score calculation. The Rad-score was calculated as a weighted linear combination of the radiomics features included in the selected model. The Rad-score and statistically significant clinical and radiological predictors were subsequently integrated to build a combined prediction model and create a nomogram.

### 2.6 Optimal Model Interpretation

To enhance the interpretability of the optimal model and mitigate the “black-box” effect, several analyses were performed. First, correlation analyses were conducted for each feature selected in the optimal radiomics model to assess the strength and direction of their association with GIST risk grade. Additionally, SHAP was used to evaluate the importance of each feature in both the optimal radiomics model and the combined model. SHAP calculates the contribution value (SHAP value) of each input feature to the predictive outcome, thereby identifying the feature importance ranking and indicating whether each feature exerts a positive or negative effect on the prediction. Both local and global interpretation results were visualized to clearly present these findings.

### 2.7 Statistical Analysis

The Kolmogorov–Smirnov test was used to assess the normality of measurement data, and the Mann–Whitney U test was used for between-group comparisons. The Chi-square test was applied for comparisons of count data. Point-biserial correlation was used to analyze correlations between each radiomics feature, the Rad-score, and the GISTs risk grade. Receiver operating characteristic (ROC) curves were constructed, and the predictive performance of the models was evaluated using the area under the curve (AUC), sensitivity (Sen), specificity (Spe), and accuracy (Acc). The model with the highest AUC in the validation set was considered the optimal model. Differences in AUCs between models were compared using DeLong's test. Calibration curve (CC) and decision curve (DC) analyses were used to assess the clinical applicability of the prediction models. The SHAP method was used to perform interpretability analysis of the optimal model. R (version 26.0), MedCalc (version 20.0), and SPSS (version 26.0) were used for statistical analyses. Two-tailed *p* < 0.05 was considered statistically significant.

## 3 RESULTS

### 3.1 Clinical and Radiological Characteristics

A total of 217 GISTs were included in this study, comprising 114 cases in the low-risk group and 103 cases in the high-risk group. Univariate analyses of clinical and radiological characteristics in the training and validation sets are presented in Table **1**. Only “maximum diameter,” “shape,” and “necrosis” showed significant differences (*p* < 0.05) between the low-risk and high-risk groups in both sets. After controlling for potential confounders using stepwise backward selection based on AIC, multivariable logistic regression revealed that maximum tumor diameter (*p* < 0.001; aOR = 2.438; 95% CI: 1.641–3.610), tumor shape (*p* = 0.029; aOR = 2.590; 95% CI: 1.101–6.094), and tumor necrosis on CT images (*p* = 0.038; aOR = 0.401; 95% CI: 0.169–0.951) remained independently associated with GIST risk classification. No significant multicollinearity was detected (all VIF values < 5; range: 1.074–1.258). These three variables were used to construct the radiological model (RM).

### 3.2 Radiomics Features and Optimal Radiomics Models

A total of 1200 radiomics features were extracted from each of the intratumoral, 3-mm peritumoral, and 5-mm peritumoral ROIs. After inter-observer reproducibility assessment, 1087, 1180, and 1174 features, respectively, remained with ICC > 0.75, indicating satisfactory reliability. The number of features with non-zero coefficients retained by the five radiomics models was as follows: 3 (ITV), 3 (PTV3), 8 (PTV5), 3 (ITV+PTV3), and 3 (ITV+PTV5). The selected features and their coefficients for each model are provided in Tables **S2–S6**, with detailed descriptions included in the Supplementary Materials.

All five radiomics models demonstrated satisfactory predictive performance. Their respective ROC curves for the training and validation sets are shown in Fig. (**3A** and **B**). In the training set, AUCs ranged from 0.890 (95% CI: 0.829–0.935) to 0.937 (95% CI: 0.886–0.970), while in the validation set, AUCs ranged from 0.861 (95% CI: 0.753–0.935) to 0.934 (95% CI: 0.843–0.980) (Table **2**). Among these, the ITV+PTV3 model exhibited the best performance in the validation set, achieving the highest AUC (0.934, 95% CI: 0.843–0.980) and accuracy (0.892). Therefore, the ITV+PTV3 model was selected as the optimal radiomics model, and its corresponding Rad-score formula is provided in the Supplementary Materials.

### 3.3 Combined Model

**Fig. (4):**
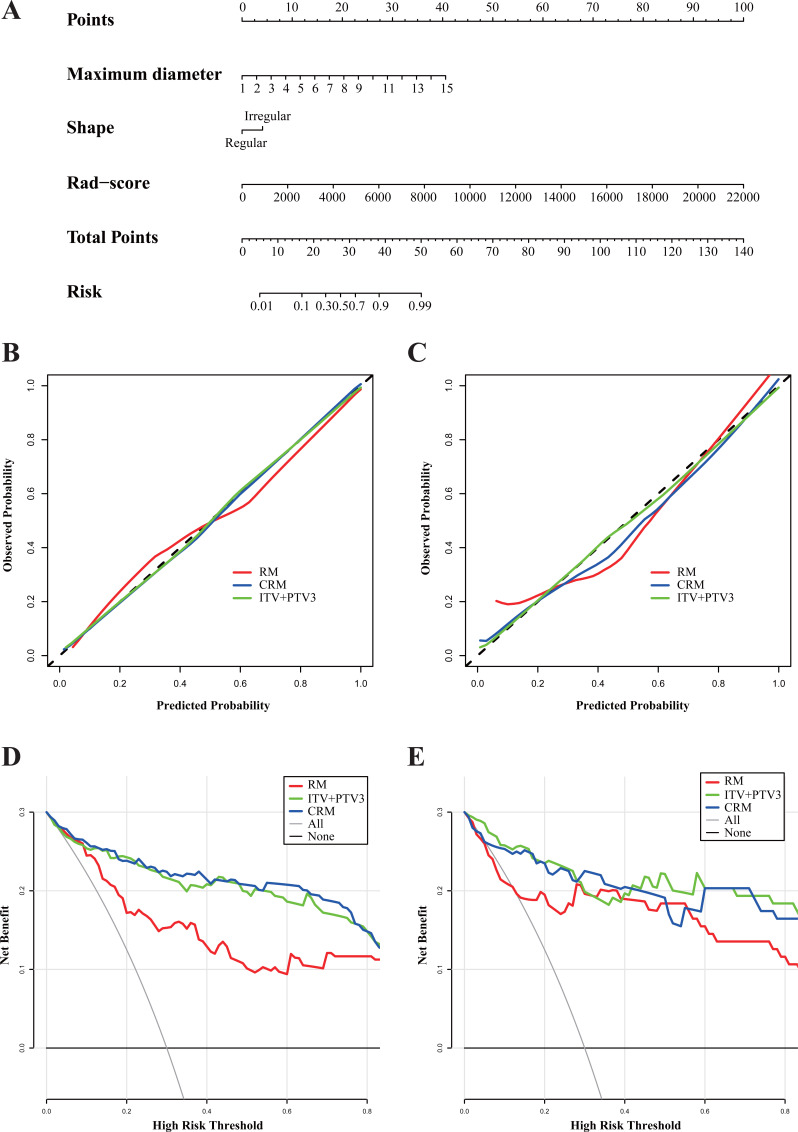
The nomogram for predicting GISTs risk-grade (**A**). Calibration curve of RM, ITV+PTV3, and nomogram in the training set (**B**) and validation set (**C**). The X-axis is the predicted probability of the outcome event, and the Y-axis is the probability of the actual occurrence of the outcome event. Decision curves for RM, ITV+PTV3, and the nomogram in the training set (**D**) and validation set (**E**). The X-axis is the threshold probability, and the Y-axis is the net benefit. CRM, combined radiological characteristics and Rad-score model. **Abbreviations: **ITV+PTV3, intratumoral combined 3-mm peritumoral model; RM, radiological characteristics model.

The selected radiological predictors were combined with the Rad-score to establish a combined model using stepwise backward logistic regression. Ultimately, “maximum tumor diameter,” “tumor shape,” and the “Rad-score” were retained in the combined model (CRM). The constructed nomogram (Fig. **4A**) illustrates that the Rad-score contributed the greatest weight to the model, followed by maximum tumor diameter.

### 3.4 Comparison of Models

The performance of RM, the optimal radiomics model (ITV+PTV3), and CRM is summarized in Table **3**. The AUCs, sensitivities, specificities, and accuracies of ITV+PTV3 and CRM showed improvements compared with RM in both the training and validation sets. Model comparison using DeLong’s test in the training set (Fig. **S2A**) and validation set (Fig. **S2B**) yielded the following findings: although the differences in AUCs between ITV+PTV3 and ITV+PTV5 were not statistically significant in either dataset, ITV+PTV3 demonstrated significantly better performance than both peritumoral models and ITV in the validation set (*p* <0.05). Additionally, ITV+PTV3 achieved higher AUC, sensitivity, specificity, and accuracy compared with CRM in the validation set, although these differences were not statistically significant. Overall, the ITV+PTV3 model emerged as the most effective among the evaluated models.

The CCs (Fig. **4B**, **C**) showed that RM, ITV+PTV3, and CRM all demonstrated good calibration, with ITV+PTV3 being closest to the ideal curve in both the training and validation sets. The DCs (Fig. **4D**, **E**) indicated that all three models achieved substantial net benefits. In the training set, across a wide range of threshold probabilities, the net benefits of ITV+PTV3 and CRM were higher than those of RM, with CRM providing a slightly greater benefit than ITV+PTV3. In the validation set, the net benefit of ITV+PTV3 exceeded that of RM except at threshold probabilities of 0.35–0.40; the net benefit of CRM exceeded RM except at 0.50–0.55; and ITV+PTV3 demonstrated a higher net benefit than CRM across most threshold ranges.

**Fig. (5):**
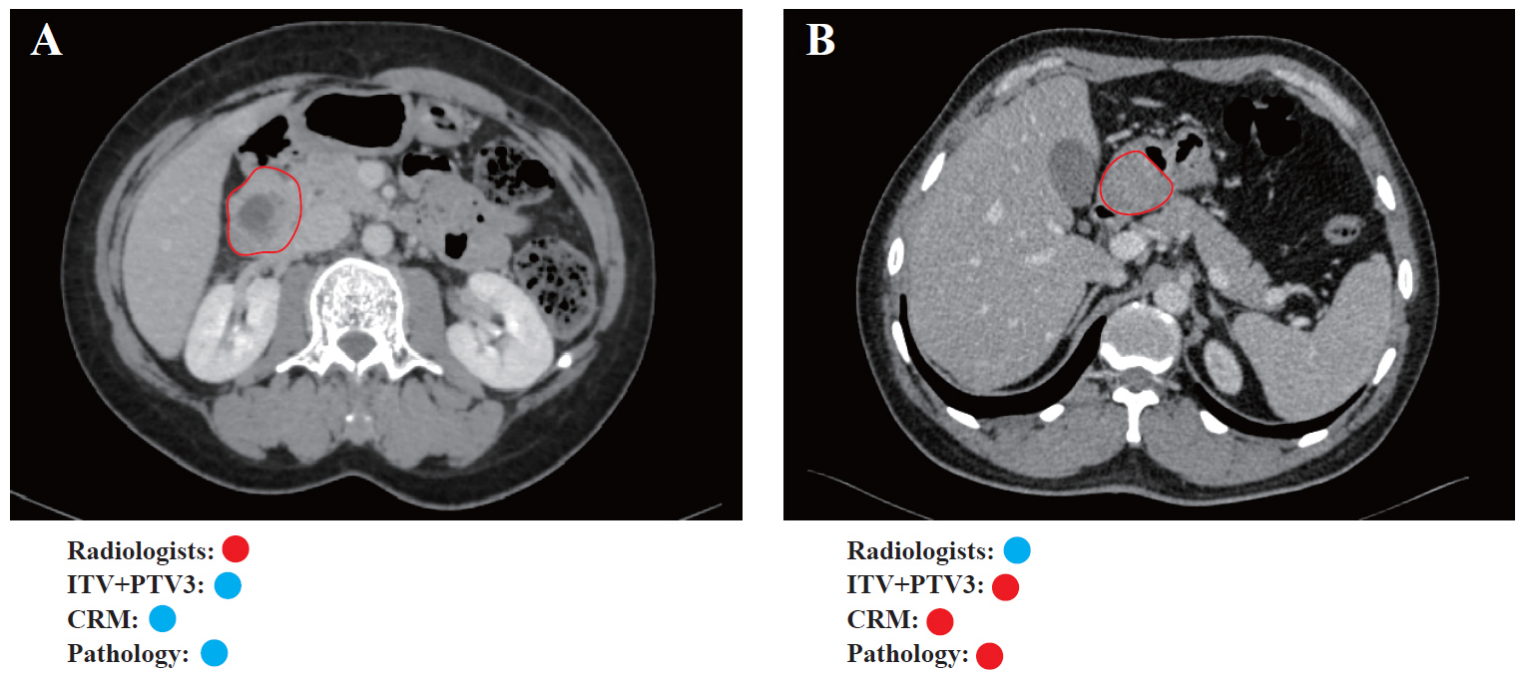
Model performance in GISTs with atypical radiological features. Red circles outline lesions on CT images. Blue dots indicate low-risk GISTs; red dots indicate high-risk GISTs. Case 1 (**A**): Low-risk GIST (mitotic count=5/50 HPFs) presenting as a 4.5 cm irregular duodenum lesion with necrosis. Rad-score=2473. Nomogram total points=24, high-risk probability=0.3. Case 2 (**B**): High-risk GIST (mitotic count=10/50 HPFs) presenting as a 4.2 cm regular-shaped intragastric lesion without necrosis. Rad-score=5376. Nomogram total points=32, high-risk probability=0.7. Both cases were misclassified by radiologists but correctly predicted by ITV+PTV3 and CRM.

We present two representative cases with atypical radiological features confirmed pathologically as low-risk (Fig. **5A**) and high-risk (Fig. **5B**) GISTs. Both cases were misclassified by two blinded radiologists (with 7 and 8 years of radiology experience, respectively) using conventional visual assessment, yet were correctly predicted by the ITV+PTV3 model (high-risk probabilities: Case 1 = 0.45; Case 2 = 0.92) and CRM (high-risk probabilities: Case 1 = 0.27; Case 2 = 0.71). These findings demonstrate the capability of radiomics to address diagnostic challenges where conventional radiological interpretation fails.

### 3.5 Interpretation of the Optimal Model

The optimal radiomics model (ITV+PTV3) incorporated the following four features: *original_shape_MajorAxisLength*, *original_shape_SphericalDisproportion*, *original_shape_ Maximum2DDiameterColumn*, and *wavelet.HLL_glszm_ GrayLevelNonUniformity*. According to the correlation analysis, these four radiomics features and the Rad-score showed a significant positive correlation between the low- and high-risk groups in the training set. These results are displayed in the violin plots in Fig. (**S3A**–**E**).

**Fig. (6):**
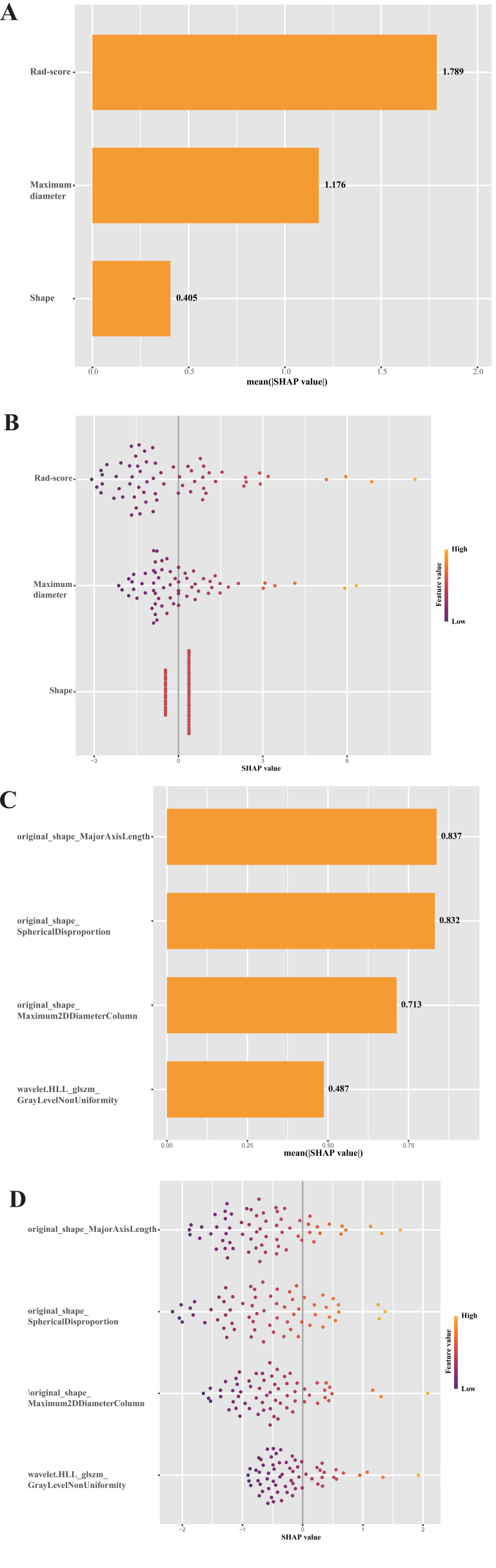
Global interpretations of the models through SHAP. Bar plot of CRM (**A**), Bee swarm plot of CRM (**B**) and ITV+PTV3 (**C**), and ITV+PTV3 (**D**). The X-axis represents the SHAP value, and the Y-axis represents input features. CRM, combined radiological characteristics and Rad-score model; ITV+PTV3, intratumoral combined 3-mm peritumoral model; SHAP, SHapley Additive Explanations.

The overall and individual SHAP values of ITV+PTV3 and the combined model (CRM) were calculated to facilitate model interpretation. The global interpretation results are shown in Fig. (**6**). The bar plot for CRM (Fig. **6A**) indicates that the Rad-score contributes the most, on average, to the model’s predictive output. The bee swarm plot for CRM (Fig. **6B**) further demonstrates that the Rad-score has a positive influence on the prediction results.

For ITV+PTV3, the bar plot (Fig. **6C**) presents the importance ranking of the four radiomics features, with *original_shape_MajorAxisLength* showing the highest average contribution, followed by *original_shape_Spherical Disproportion*. The bee swarm plot (Fig. **6D**) illustrates the distribution of SHAP values across all samples, showing that high feature values (red) tend to contribute positively to the prediction, whereas low values (purple) tend to contribute negatively.

**Fig. (7):**
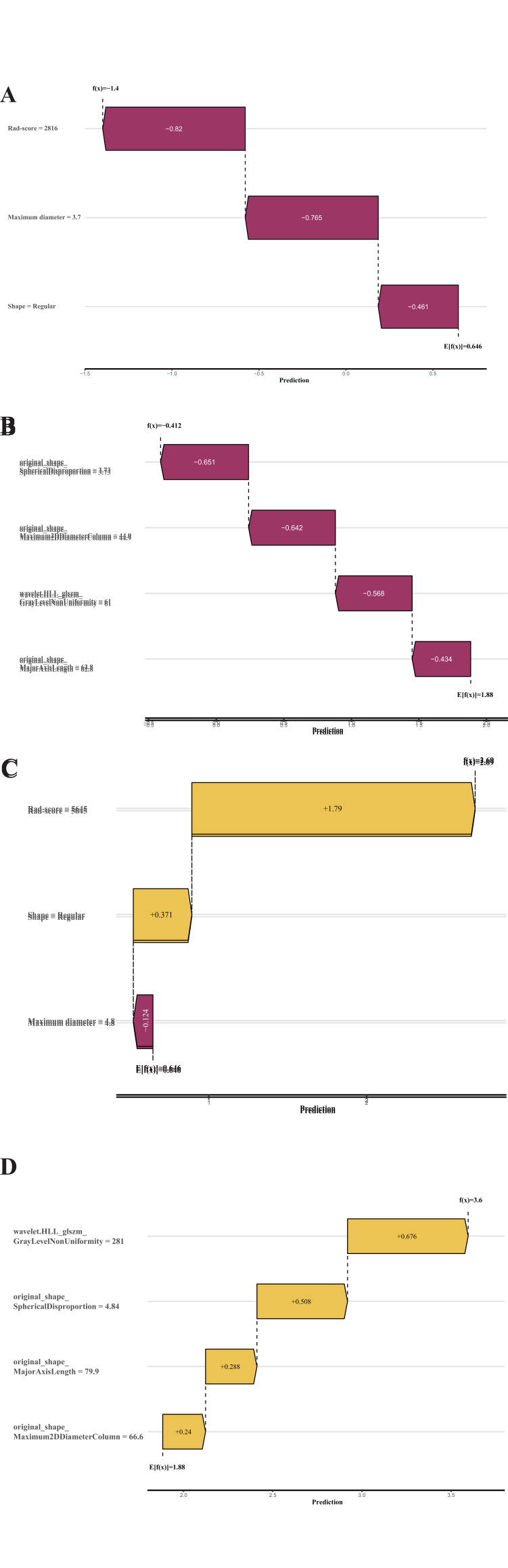
Local interpretations of the models through SHAP. Waterfall plots of SHAP values for a low-risk grade sample, generated by the CRM (**A**) and ITV+PTV3 (**B**). Waterfall plots of SHAP values for a high-risk grade sample, generated by the CRM (**C**) and ITV+PTV3 (**D**). The X-axis represents the SHAP value, and the Y-axis represents input features. CRM, combined radiological characteristics and Rad-score model; ITV+PTV3, intratumoral combined 3-mm peritumoral model SHAP, SHapley Additive Explanations.

Local interpretation is shown in Fig. (**7**). A representative low-risk sample was evaluated using both CRM (Fig. **7A**) and ITV+PTV3 (Fig. **7B**), while a representative high-risk sample was evaluated using CRM (Fig. **7C**) and ITV+PTV3 (Fig. **7D**). The waterfall plots visually demonstrate how each feature, through its positive or negative contribution, influenced the final model prediction.

## 4 DISCUSSION

GISTs exhibit complex biological behavior and varying levels of aggressiveness. Accurate risk grading is essential for individualized treatment. However, the risk-grade systems currently used in clinical practice remain insufficient. In this study, we established multiple models to predict the risk classification of GISTs using radiomics features extracted from both intratumoral and peritumoral regions, and further visualized the prediction process using SHAP. Among the developed models, ITV+PTV3 demonstrated the best performance in the validation set and can serve as a reliable assessment tool for GIST risk grading.

Several studies have investigated the association between clinical and radiological characteristics and the risk grade of GISTs. In our univariate analysis, gastrointestinal bleeding did not differ significantly between the low-risk and high-risk groups, which contrasts with findings from previous studies [24, 25]. We identified “maximum diameter,” “shape,” and “necrosis” as significant radiological predictors of GIST risk grade and constructed a radiological characteristics model (RM) accordingly. Consistent with most prior studies [26, 27], maximum tumor diameter was a key feature that differed significantly between the groups. Other studies have reported associations between tumor location, margin, growth pattern, ulceration, or calcification and GIST risk [28, 29]; however, these relationships were not observed in our study, possibly due to the subjectivity inherent in radiological assessment. As illustrated in Fig. (**5A**, **B**), the radiomics model performed robustly even in morphologically ambiguous cases, suggesting its potential to reduce inter-observer variability among radiologists. Furthermore, the combined model (CRM) incorporated the Rad-score, maximum tumor diameter, and tumor shape, with the Rad-score contributing the greatest weight.

Although CRM showed improved performance compared to RM, its predictive accuracy was not significantly different from that of the other radiomics models, including the ITV+PTV3 model. This finding suggests that adding radiological characteristics to the radiomics model may not substantially enhance the predictive performance for GIST risk stratification. Previous studies have also confirmed the strong predictive capability of radiomics for GIST risk classification [30, 31]. However, these studies did not investigate the value of peritumoral radiomics. In the present study, we examined both intratumoral and peritumoral regions, and the results demonstrated that incorporating peritumoral regions provides a more comprehensive assessment of GIST risk classification.

Researchers have proposed that radiomics features extracted from peritumoral regions can capture subtle microenvironmental changes and may serve as reliable biomarkers [32, 33]. To the best of our knowledge, only one study to date has constructed a radiomics model based on peritumoral regions to predict GIST risk classification, reporting that peritumoral regions outperformed intratumoral regions in prediction [34]. There are several differences between that study and ours. First, we used full-layer segmentation rather than maximum-level delineation, as whole-volume segmentation can more comprehensively capture the characteristics of a lesion. Additionally, we found that in the training set, the AUCs of both the peritumoral model and the combined intratumoral–peritumoral model were higher than that of the intratumoral model (*p* <0.05). In the validation set, although no significant difference was observed between the AUCs of the intratumoral and peritumoral models, the combined intratumoral + 3-mm peritumoral model outperformed all single-region models. We believe that intratumoral and peritumoral regions each reflect different biological information and may vary in predictive importance depending on tumor type and clinical context, yet both possess distinct clinical value. Their combined use offers a more comprehensive representation of tumor biology.

The optimal peritumoral range remains controversial in peritumoral radiomics studies. Based on relevant research [35, 36], we selected 3-mm and 5-mm peritumoral ranges for radiomics feature extraction. We found that PTV3 had higher AUC values than PTV5, although the differences were not statistically significant; the 3-mm peritumoral features also performed better when combined with intratumoral features. This finding suggests that the 3-mm peritumoral region may contain more relevant information for GIST risk stratification compared with the 5-mm range. We assume that because the surrounding structures of GISTs are often complex, this complexity may contribute to greater variation in feature values; however, the amount of information directly related to tumor invasion may be relatively limited, potentially affecting the model’s reliability and stability. Therefore, smaller peritumoral ranges performed better in this study. This observation warrants further confirmation through more precise peritumoral range definition and a larger sample size.

To facilitate the clinical translation of radiomics models, correlation analysis and the SHAP method were employed to enhance interpretability. Among the four features of ITV+PTV3, “original_shape_MajorAxisLength” exhibited the highest SHAP value. This feature represents the maximum diameter of the ROI in 3D space, which aligns with the NIH criteria in pathological assessments [3]. Since tumor size is a key component of GIST risk grading, larger lesions typically indicate higher risk. “Original_shape_SphericalDisproportion” was the second most important feature and showed a positive correlation with model predictions. Mathematically, a higher value indicates greater morphological irregularity, which pathologically reflects the tendency of more aggressive tumors to breach surrounding tissue barriers, resulting in irregular shapes. Our findings also indicate that higher values of “original_shape_Maximum2DDiameterColumn” correspond to an increased risk of GISTs. This feature quantifies the vertical dimension of lesion extension and may reflect the tumor's growth pattern and the presence of anisotropic growth.

“Wavelet.hll_glszm_GrayLevelNonUniformity” is a texture feature derived through wavelet transformation. It indicates potential intratumoral heterogeneity, such as necrosis or calcification, which causes inhomogeneity in gray-level distribution. Our analysis shows that this feature is positively correlated with GIST risk. Previous studies have reported that GIST heterogeneity is associated with increased recurrence risk and poorer survival outcomes [37]. Numerous studies have confirmed that pathological alterations within both intratumoral and peritumoral regions constitute the biological basis of radiomics features [38, 39]. The objective, quantitative, and reproducible radiomics features we selected for predicting GIST risk grade, therefore, exhibit clear biological plausibility. Nevertheless, tumor pathology is complex and subtle, and the precise correspondence between radiomics features and specific pathological changes in GISTs requires further investigation using larger samples and rigorous pathological correlation.

## 5 STUDY LIMITATION

Our study has several limitations. Firstly, it was a single-center retrospective study, and the conclusions must be validated prospectively across multiple centers to enhance the generalizability of the model. Secondly, only venous-phase images were used to clearly visualize tumor boundaries. Whether plain and arterial-phase scans may provide additional useful information remains to be further explored. Thirdly, although we preliminarily investigated the relationship between radiomics features and the risk grade of GISTs, future studies should incorporate precise image–pathology comparisons to better elucidate the biological basis of radiomic features. Finally, to balance the sample size, we categorized cases into low-risk and high-risk groups. Larger datasets and multi-classification studies will be needed in the future to meet actual clinical requirements.

## CONCLUSION

In conclusion, a combined intratumoral and 3-mm peritumoral radiomics risk-stratification model was developed, which can effectively predict the risk grade of GISTs. This model may serve as a non-invasive, objective, and reproducible biomarker to support clinical prognosis evaluation and individualized treatment planning for patients with GISTs.

## Figures and Tables

**Fig. (1):**
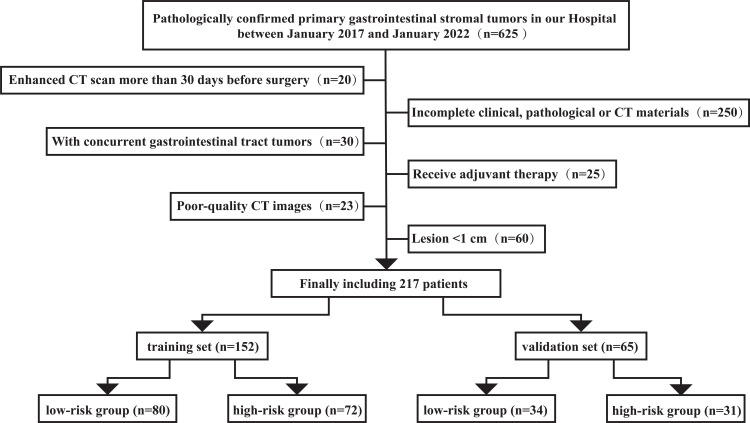
The process of screening patients.

**Fig. (2):**
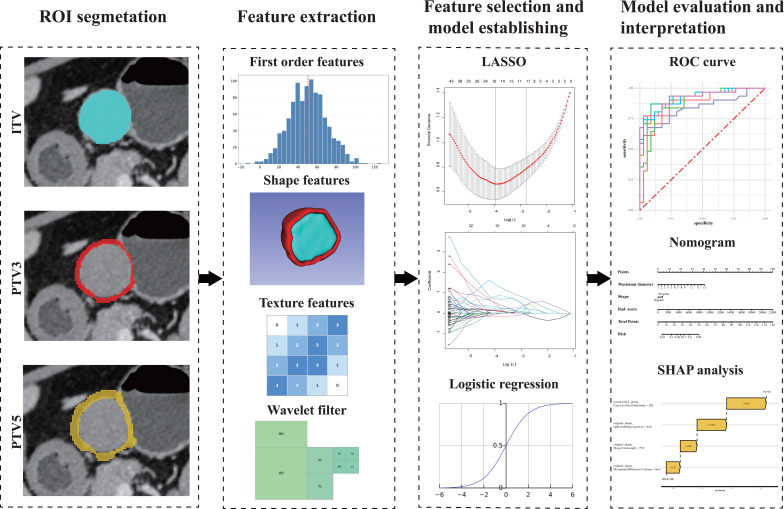
The process of radiomics in this study, including: ROI segmentation, feature extraction, feature selection, model establishment, model evaluation, and interpretation. CRM, combined radiological characteristics and Rad-score model; ITV, intratumoral model; ITV+PTV3, intratumoral combined 3-mm peritumoral model; ITV+PTV5, intratumoral combined 5-mm peritumoral model; Lasso, least absolute shrinkage and selection operator; PTV3, 3-mm peritumoral model; PTV5, 5-mm peritumoral model; RM, radiological characteristics model; ROC, receiver operating characteristic; ROI, region of interest; SHAP, SHapley Additive Explanations.

**Fig. (3):**
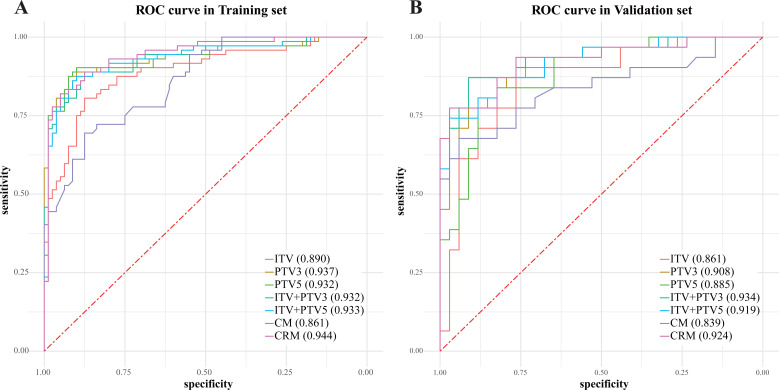
The ROC curves of different models in the training set (**A**) and validation set (**B**). The X-axis represents specificity, and the Y-axis represents sensitivity. CRM, combined radiological characteristics and Rad-score model. **Abbreviations: **ITV, intratumoral model; ITV+PTV3, intratumoral combined 3-mm peritumoral model; ITV+PTV5, intratumoral combined 5-mm peritumoral model; PTV3, 3-mm peritumoral model; PTV5, 5-mm peritumoral model; RM, radiological characteristics model; ROC, receiver operating characteristic.

**Table 1:** Univariate analysis results for clinical and radiological characteristics of the training and validation sets.

Characteristics	Training Set(n=152)		*p*-value	Validation set(n=65)		*p*-value
	Low-risk Group (n=80)	High-risk group (n=72)		Low-risk Group (n=34)	High-risk Group (n=31)	
Age, years (Mean±SD)	58.2±13.0	59.2±13.1	0.542	60.3±11.0	62.5±14.1	0.337
Sex [n (%)]			0.392			0.730
Male	40(50.0%)	41(56.9%)		19(55.9%)	16(51.6%)	
Female	40(50.0%)	31(43.1%)		15(44.1%)	15(48.4%)	
Digestive tract bleeding	55(68.8%)	42(58.3%)	0.182	17(50.0%)	7(22.6%)	0.022
Maximum diameter, cm (Mean±SD)	3.5±1.0	6.6±3.2	<0.001	3.7±1.2	6.6±3.3	<0.001
Tumor location [n (%)]			0.820			0.260
stomach	58(72.5%)	51(70.8%)		22(64.7%)	24(77.4%)	
non-stomach	22(27.5%)	21(29.2%)		12(35.3%)	7(22.6%)	
Shape [n (%)]			0.001			0.005
Regular	46(57.5%)	22(30.6%)		18(52.9%)	6(19.4%)	
Irregular	34(42.5%)	50(69.4%)		16(47.1%)	25(80.6%)	
Boundary [n (%)]			1.000			0.951
Clear	60(75.0%)	54(75.0%)		25(73.5%)	23(74.2%)	
Unclear	20(25.0%)	18(25.0%)		9(26.5%)	8(25.8%)	
Growth Pattern [n (%)]			0.286			0.105
Endogenous growth	27(33.8%)	16(22.2%)		15(44.1%)	9(29.0%)	
Exogenous growth	32(40.0%)	33(45.8%)		7(20.6%)	14(45.2%)	
Mixed growth	21(26.3%)	23(31.9%)		12(35.3%)	8(25.8%)	
Calcification [n (%)]	8(10.0%)	15(20.8%)	0.063	4(44.4%)	5(16.1%)	0.611
Necrosis [n (%)]	20(25.0%)	45(62.5%)	<0.001	12(35.3%)	18(58.1%)	0.066
Ulcer [n (%)]	16(20.0%)	22(30.6%)	0.133	9(26.5%)	4(12.9%)	0.172

**Note: **SD, standard deviations.

**Table 2:** The performance of radiomics models.

Model	Training Set (n=152)				Validation Set (n=65)			
	AUC (95% CI)	Sen	Spe	Acc	AUC (95% CI)	Sen	Spe	Acc
ITV	0.890(0.829-0.935)	0.806	0.875	0.842	0.861(0.753-0.935)	0.903	0.735	0.815
PTV3	0.937(0.886-0.970)	0.875	0.900	0.888	0.908(0.810-0.966)	0.807	0.912	0.815
PTV5	0.932(0.880-0.967)	0.903	0.900	0.901	0.885(0.782-0.951)	0.807	0.882	0.846
ITV+PTV3	0.932(0.891-0.973)	0.890	0.875	0.882	0.934(0.843-0.980)	0.871	0.912	0.892
ITV+PTV5	0.933(0.891-0.975)	0.875	0.900	0.888	0.919(0.854-0.985)	0.742	0.971	0.862

**Note: **Acc, accuracy; AUC, area under the curve; CI, confidence interval; ITV, intratumoral model; ITV+PTV3, intratumoral combined 3-mm peritumoral model; ITV+PTV5, intratumoral combined 5-mm peritumoral model; PTV3, 3-mm peritumoral model; PTV5, 5-mm peritumoral model; Sen, sensitivity; Spe, specificity.

**Table 3:** The performance of radiological, optimal radiomics, and combined models.

Model	Training Set (n=152)				Validation Set (n=65)			
	AUC (95% CI)	Sen	Spe	Acc	AUC (95% CI)	Sen	Spe	Acc
RM	0.861(0.795-0.912)	0.722	0.825	0.776	0.839(0.727-0.919)	0.710	0.765	0.739
ITV+PTV3	0.932(0.891-0.973)	0.890	0.875	0.882	0.934(0.843-0.980)	0.871	0.912	0.892
CRM	0.944(0.895-0.975)	0.833	0.913	0.875	0.924(0.831-0.975)	0.839	0.824	0.831

**Note: **Acc, accuracy; AUC, area under the curve; CI, confidence interval; CRM, combined radiological characteristics and Rad-score model; ITV+PTV3, intratumoral combined 3-mm peritumoral model; RM, radiological characteristics model; Sen, sensitivity; Spe, specificity.

## Data Availability

All the data and supporting information are provided within the article.

## LIST OF ABBREVIATIONS

GISTs: Gastrointestinal stromal tumors
SHAP: Shapley Additive Explanations
RM: Radiological model
CT: Computed tomography
AIC: Akaike information criterion
VIF: Variance inflation factors

## STANDARDS OF REPORTING

STROBE guidelines were followed.
