# Supplementary material for: Intratumoral and Peritumoral Radiomics based on Contrast-enhanced CT Images: Biomarkers to Predict the Risk-Grade of Gastrointestinal Stromal Tumors
Source: Curr Med Imaging. 2026 Feb 2;22:e15734056394776. doi: 10.2174/0115734056394776251126063546 (PMC13445552; doi:10.2174/0115734056394776251126063546)
Supplement: Supplementary file 1 [file CMIM-22-E15734056394776_SD1.pdf]

1875-6603/26

Send Orders for Reprints to reprints@benthamscience.net

BENTHAM SCIENCE

CrossMark

Current Medical Imaging

Content list available at: https://benthamscience.com/journals/cmir

Supplementary Material

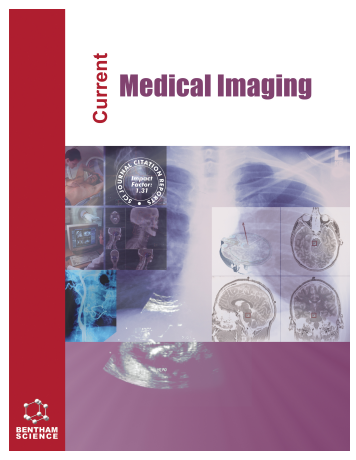

Intratumoral and Peritumoral Radiomics based on Contrast-enhanced CT Images: Biomarkers to Predict the Risk-Grade of Gastrointestinal Stromal Tumors

Yiming Liu[1,2], Ying Wu[2], Xinyue Cao[2], Guojin Zhang[2] and Longlin Yin[2,3,*]

[1]*Department of Radiology, Sichuan Provincial People's Hospital, Wenjiang Hospital, Chengdu, China*
[2]*Department of Radiology, Sichuan Provincial People's Hospital, University of Electronic Science and Technology of China, Chengdu, China*
[3]*Institute of Radiation Medicine, Sichuan Provincial People's Hospital, University of Electronic Science and Technology of China, Chengdu, China*

Table S1. Risk classification criteria of primary GISTs after resection (NIH 2008 modified version).

Risk Category	Tumor Size (cm)	Mitotic Index (per 50 HPFs)	Primary Tumor Site
Very low risk	<2.0	≤5	Any
Low risk	2.1-5.0	≤5	Any
Intermediate risk	2.1-5.0	>5	Gastric
-	<5.0	6-10	Any
-	5.1-10.0	≤5	Gastric
High risk	Any	Any	Tumor rupture
-	>10	Any	Any
-	Any	>10	Any
-	>5.0	>5	Any
-	2.1-5.0	>5	Nongastric
-	5.1-10.0	≤5	Nongastric

Table S2. The selected features and their coefficients of ITV.

Name	Category	Coeffcient
original_shape_SurfaceVolumeRatio	Shape	-0.486667358
log-sigma-4-mm-3D_glrlm_RunEntropy	GLRLM	0.856801289
log-sigma-4-mm-3D_glrlm_RunVariance	GLRLM	0.833056924

Table S3. The selected features and their coefficients of PTV3.

Name	Category	Coeffcient
original_shape_SphericalDisproportion	Shape	2.364683217
original_glszm_GrayLevelNonUniformity	GLSZM	0.009236167
log.sigma.4.mm.3D_glcm_Idn	GLCM	6.88346092

DOI: 10.2174/0115734056394776251126063546, 2026, *22*, e15734056394776

Table S4. The selected features and their coefficients of PTV5.

Name	Category	Coeffcient
original_shape_Sphericity	Shape	-1.444508225
log-sigma-2-mm-3D_glszm_GrayLevelNonUniformity	GLSZM	0.049824761
log-sigma-4-mm-3D_glcm_Idn	GLCM	0.307181513
wavelet-LHL_glszm_SizeZoneNonUniformity	GLSZM	0.409175218
wavelet-LHL_glrlm_RunLengthNonUniformity	GLRLM	0.899458284
wavelet-LHH_glszm_GrayLevelNonUniformity	GLSZM	0.069907676
wavelet-HLL_gldm_GrayLevelNonUniformity	GLDM	0.153165519
wavelet-HLL_glszm_LargeAreaHighGrayLevelEmphasis	GLSZM	0.467366195

Table S5. The selected features and their coefficients of ITV+PTV3.

Name	Category	Region of Interest	Coeffcient
original_shape_MajorAxisLength	Shape	PTV3	1.599566577
original_shape_SphericalDisproportion	Shape	PTV3	1.397375204
original_shape_Maximum2DDiameterColumn	Shape	ITV	1.261910905
wavelet.HLL_glszm_GrayLevelNonUniformity	GLSZM	PTV3	-0.384006423

Table S6. The selected features and their coefficients of ITV+PTV5.

Name	Category	Region of Interest	Coeffcient
original_shape_Maximum2DDiameterColumn	Shape	ITV	2.057858893
wavelet-HLL_ngtdm_Coarseness	NGTDM	PTV5	-1.002397999
original_shape_Maximum2DDiameterRow	Shape	ITV	0.855466192
original_shape_MajorAxisLength	Shape	PTV5	0.690437618
original_shape_Sphericity	Shape	PTV5	-0.057986213

The detailed definitions of radiological characteristics are as follows:

When the tumor was lobulated, the shape of the tumor was deemed irregular. When a tumor grew into the stomach or intestines' lumen, it was classified as endogenous growth; when it expanded outside the serosal surface, it was classified as exogenous growth; and when the tumor crossed the stomach or intestines' wall and simultaneously grew intraluminally and extraluminally, it was classified as mixed growth. A restricted defect on the tumor surface was deemed indicative of ulceration. When a local CT value of greater than 100 HU was seen in the tumor on CT plain images, calcification was deemed to be present. Necrosis was deemed to be present in tumor with a plain scan CT value of 0 to 30 HU and an enhancement CT value increase of no more than 5 HU.

The explanation of each selected feature:

This study adopts internationally recognized standards for radiomics feature extraction. Feature names follow a hierarchical naming convention in the format of "ImageType_FeatureClass_FeatureName". The prefix "original" indicates the features extracted from the original image. The "log" indicates features extracted from images transformed *via* Laplacian of Gaussian (LoG) filtering, where "sigma-4-mm" specifies the filter parameter ($\sigma = 4$ mm), and "3D" denotes the filtering was performed on three-dimensional (3D) data. The "wavelet" indicates extraction from images transformed *via* wavelet filtering. The three-letter following "wavelet" encodes the frequency band combination of 3D wavelet decomposition (each letter corresponds to a direction: row, column, and slice; *e.g.*, ‘LHL’ denotes Low-Row, High-Column, Low-Slice). The "FeatureClass" includes shape features and various texture features. Shape features mainly describe the geometric shape, size, and boundary characteristics of the region of interest (ROI). Texture features such as Gray Level Size Zone Matrix (GLSZM) quantify the texture heterogeneity of ROI by analyzing the joint distribution of "gray level" and "region size" in the image. The "FeatureName" indicates the specific name of the feature within its class. To facilitate precise interpretation, we selected four features from the optimal radiomics model ITV+PTV3 for further explanation:

1. MajorAxisLength

It represents the major axis length of the ROI, serving as a key indicator to describe the 3D lesion morphology and belonging to shape features. It is calculated by principal component analysis:

$$MajorAxisLength = 4\sqrt{\lambda max}$$

It quantifies the maximum extension distance of the tumor in physical space, and a higher value indicates a wider local infiltration range.

2. SphericalDisproportion

It represents the degree to which the 3D shape of ROI deviates from the perfect sphere, belongs to shape features, and its calculation formula is as follows:

$$SphericalDisproportion = \frac{Vsphere}{V}$$

It quantifies the irregularity of the tumor; higher values indicate more irregular tumor morphology (*e.g.*, lobulation, speculation), suggesting adverse biological behavior such as invasive tumor growth.

3. Maximum2DDiameterColumn

It represents the maximum extension of the ROI in the vertical direction and belongs to shape features. Its calculation formula is as follows:

$$Maximum2DDiameterColumn = \max_{z}(\text{Max2DDiameter}_z)$$

It quantifies the maximum width variation of the tumor across different planes. Combined analysis with diameters in other directions helps identify irregular expansion or deformation of the tumor, reflecting the tumor's growth pattern and its relationship with adjacent structures.

4. GrayLevelNonUniformity

It represents the consistency of frequency distribution of different grey values within the ROI, and is an important texture feature in the Grey Level Run Length Matrix (GLRLM). Its calculation formula is as follows:

$$GrayLevelNonUniformity = \sum_{i=1}^{N_g}\left(\frac{f_i}{N_p}\right)^2$$

It quantifies the spatial inhomogeneity of gray value distribution within the tumor. Higher values indicate greater intensity heterogeneity, directly reflecting increased compositional complexity in tumor tissues (*e.g.*, co-existence of necrotic, hemorrhagic, and calcified components).

Rad-score calculation formula

Rad-score = 0.966947389 + 1.599566577 * PTV3 original shape Major Axis Length + 1.397375204 * PTV3 original shape Spherical Disproportion + 1.261910905 * ITV original shape Maximum 2D Diameter Column - 0.384006423 * PTV3 wavelet HLL glszm Gray Level NonUniformity.

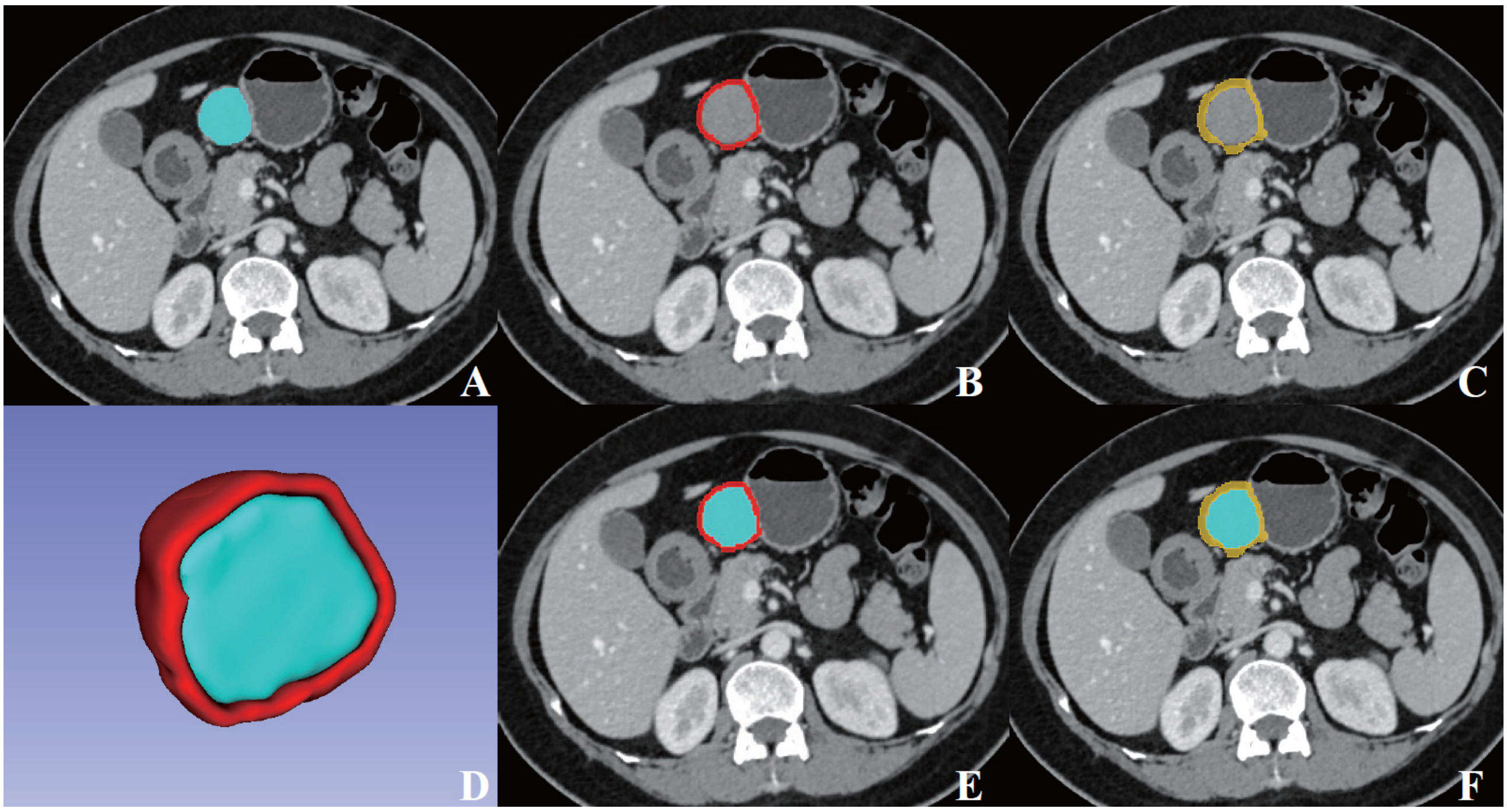

Fig. (S1). (**A-F**) Schematic diagram of intratumoral and peritumoral ROI segmentation. The example segmentation was from a 50-year-old woman diagnosed with a gastric stromal tumor, low risk according to the NIH 2008 modified version. The blue masks were the intratumoral ROIs (**A**, **D-F**). The red masks were the 3-mm peritumoral ROIs (**B**, **E**, **D**). The yellow masks were the 5-mm peritumoral ROIs (**C**, **F**).

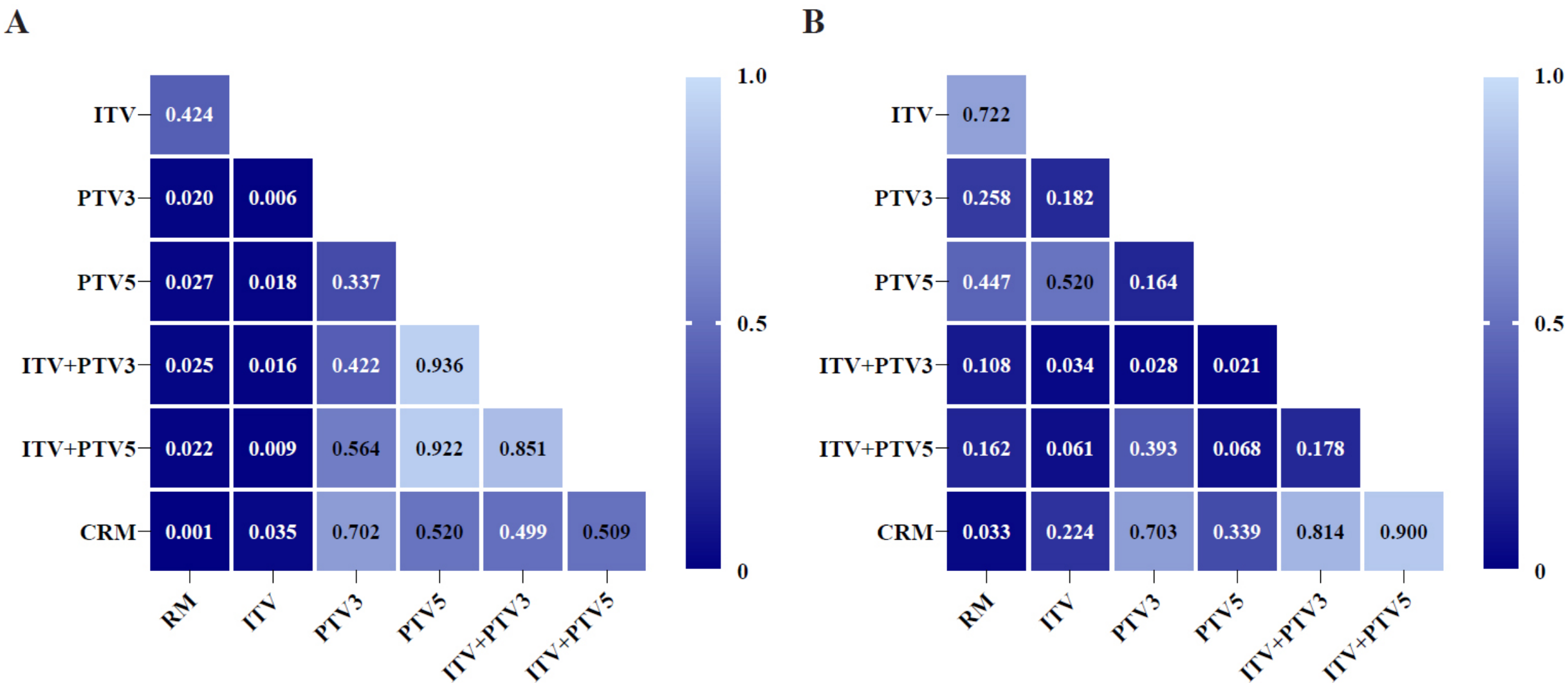

Fig. (S2). Heatmaps of pairwise comparison *via* Delong's test in the training set (**A**) and validation set (**B**). The *p*-values are presented as grid heatmaps, with dark blue indicating statistical differences (p<0.05). CRM, combined radiological characteristics and Rad-score model; ITV, intratumoral model; ITV+PTV3, intratumoral combined 3-mm peritumoral model; ITV+PTV5, intratumoral combined 5-mm peritumoral model; PTV3, 3-mm peritumoral model; PTV5, 5-mm peritumoral model; RM, radiological characteristics model.

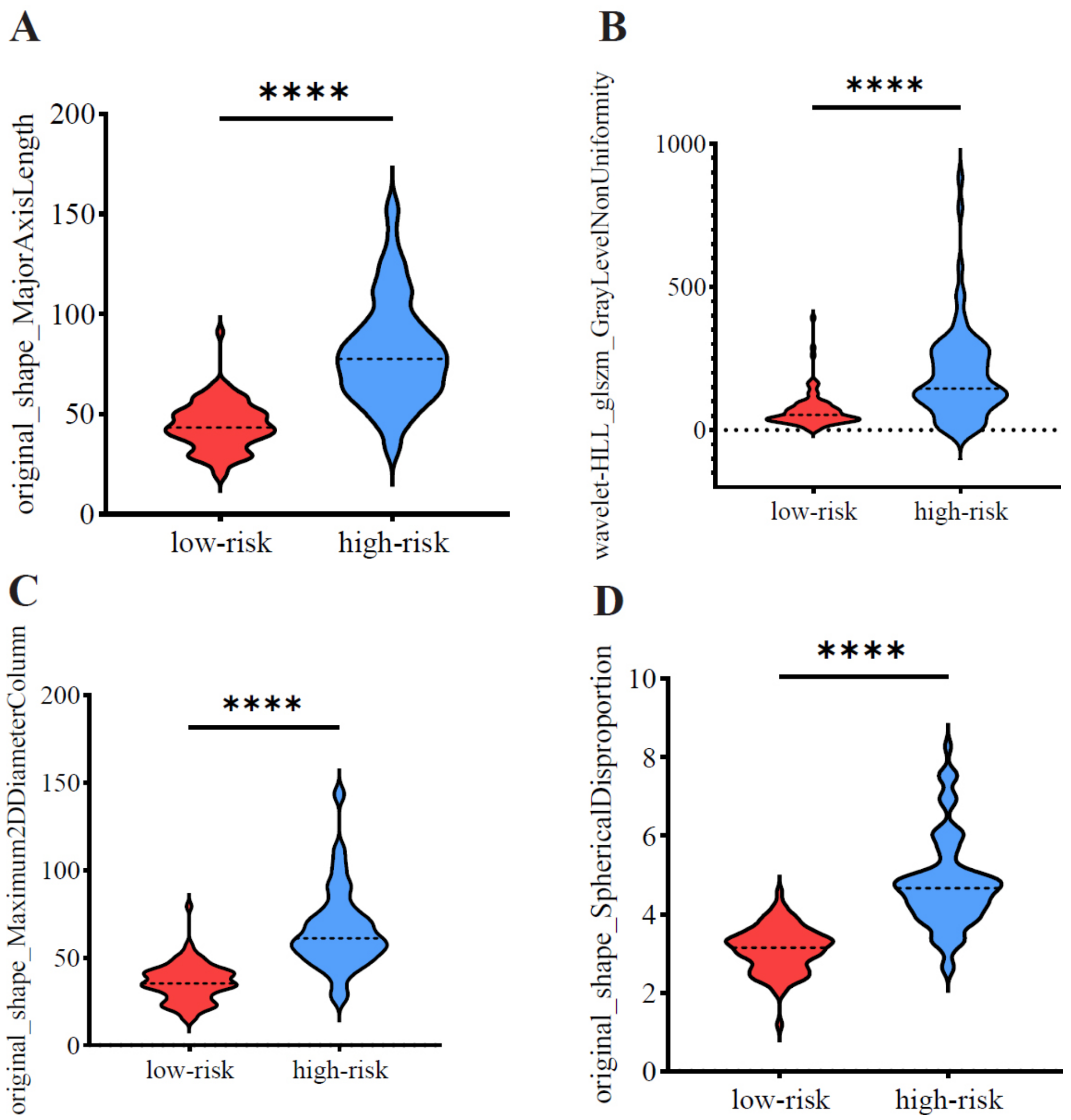

Fig. S3 contd.....

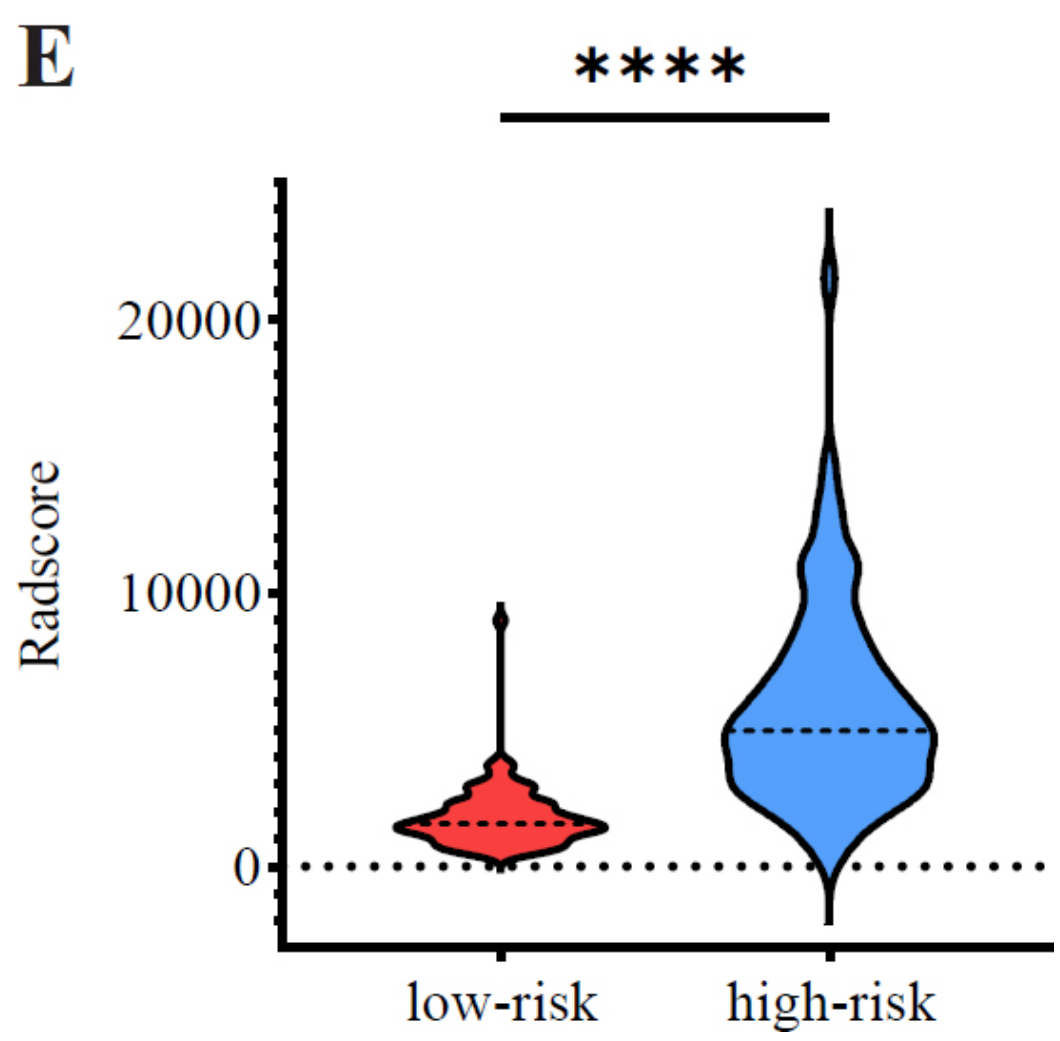

Fig. (S3). Violin plots showed four radiomics features "original_shape_MajorAxisLength" (**A**), "wavelet.HLL_glszm_GrayLevelNonUniformity" (**B**), "original_shape_Maximum2DDiameterColumn" (**C**), "original_shape_SphericalDisproportion" (**D**), and the Rad-score (**E**) were significantly different between the low-risk and high-risk groups in the training set (****$p < 0.001$).

© 2026 The Author(s). Published by Bentham Science Publisher.

This is an open access article distributed under the terms of the Creative Commons Attribution 4.0 International Public License (CC-BY 4.0), a copy of which is available at: https://creativecommons.org/licenses/by/4.0/legalcode. This license permits unrestricted use, distribution, and reproduction in any medium, provided the original author and source are credited.

HOW TO CITE:
Liu Y, Wu Y, Cao X, Zhang G, Yin L. Intratumoral and Peritumoral Radiomics based on Contrast-enhanced CT Images: Biomarkers to Predict the Risk-Grade of Gastrointestinal Stromal Tumors. Curr Med Imaging, 2026; 22: e15734056394776.
http://dx.doi.org/10.2174/0115734056394776251126063546
